# The Longitudinal Examination of Associations Among Coping Styles, Sociocultural Context, and Re-Entry Stress After Study Abroad

**DOI:** 10.1177/01461672251391059

**Published:** 2025-12-01

**Authors:** Adela Černigoj, Paul E. Jose, Ágnes Szabó, Nicolas Geeraert

**Affiliations:** 1 Victoria University of Wellington, New Zealand; 2 University of Essex, Colchester, UK

**Keywords:** reverse culture shock, reacculturation, cultural adaptation, cultural values, stress and coping

## Abstract

This research explored how approach, acceptance, and avoidance coping styles predicted re-entry stress. Second, it examined how home country sociocultural factors (individualism/collectivism, flexibility/monumentalism, cultural heterogeneity, and Human Development Index) predicted re-entry stress. Third, interaction effects between coping styles and country-level variables on re-entry stress were explored. We analyzed data from an 18-month longitudinal study which followed 1485 high school students before going abroad, while staying abroad, and after returning home. Students came from 45 home countries and studied abroad for 8–10 months. When controlling for baseline levels of stress, multilevel modeling analyses showed that acceptance coping predicted lower re-entry stress while avoidance coping predicted greater re-entry stress. Participants returning home to countries with higher collectivism and cultural homogeneity experienced greater re-entry stress. Collectivism moderated the effect of approach coping on re-entry stress, so that coping by not approaching the stressor was associated with greater stress in collectivist cultures.

## Introduction

In 1673, Descartes (2017) wrote, “when one takes too much time traveling, one eventually becomes a stranger in one’s own country” (part I, p. 4), acknowledging difficulties one might face when returning back to their home country after staying abroad. The International Organization for Migration (IOM, 2024) estimated that there were around 281 million international immigrants worldwide in 2020. An important proportion of international immigrants are temporary expatriates, who reside in another county for a limited period of time and eventually return home. According to Finnacord (2018), there were more than 66 million temporary expatriates worldwide in 2017. This number has been rising at a compound annual rate of over 5% since 2013, with student sojourners being the second largest group of temporary expatriates (after workers). Previous studies have found that student sojourners who return to their home countries often report heightened levels of stress and lower well-being (Alkhalaf, 2019; Dykhouse & Bikos, 2019; Gray & Savicki, 2015), including feelings of disorientation, alienation, and depression (Kartoshkina, 2015). Re-entry stress, sometimes also referred to as “reverse culture shock” (Gaw, 2000; Presbitero, 2016) or “acculturative stress on return” (Geeraert & Ward, 2025) can have detrimental effects on health and well-being. In light of the increase of temporary stays abroad due to education, it is important to understand how re-entry into the home culture impacts students’ stress levels and what individual and sociocultural factors play a role in this process.

## Literature Review

When people move to a foreign country, they may expect to face difficulties related to cultural differences, language barriers, unfamiliar habits, or food. However, when sojourners return to their home country, they are often unaware of the fact that they themselves have changed, or that the home environment and people have changed in the time of their absence. The lack of realistic expectations may cause unanticipated stress and difficulties with re-adapting (Geeraert et al., 2022; Gullahorn & Gullahorn, 1963). Depending on how people respond to or cope with the challenges and demands of the re-entry process, they may experience heightened levels of re-entry stress (Ward, 2001). Surprisingly, the reacculturation literature has largely neglected the role of coping in managing re-entry stress. A recent systematic literature review on reacculturation (Černigoj et al., 2024) identified only one study that quantitatively explored the relationship between coping strategies and re-entry adjustment (Herman & Tetrick, 2009). We address this gap in the literature by examining whether approach, avoidance, and acceptance coping styles predict re-entry stress.

### The Stress and Coping Framework

Coping styles are an important individual difference variable in managing stressful life changes. Lazarus and Folkman’s (1984) typology of coping styles distinguishes between problem-focused strategies and emotion-focused strategies. Problem-focused strategies are directed toward proactively changing the stressor and taking action when faced with a stressful situation (i.e., approaching the stressor). Emotion-focused strategies, however, are directed toward regulating/reducing emotional distress caused by a stressor (e.g., fear, sadness, and anger), rather than changing the stressor itself. One can strive to regulate emotional distress in two main ways, either by *dealing* with a stressor, for example, changing one’s view on the situation or accepting the situation, or by *avoiding to deal* with a stressor, for example, denial or avoidance. Related to this typology, we investigate three coping styles, that is, approach, acceptance, and avoidance, and their associations with re-entry stress. While little literature is available in relation to re-entry stress, we based our predictions on studies exploring a diverse set of situations and stressors. Studies have generally found that acceptance and approach coping styles are adaptive strategies in reducing stress while avoidance has typically been considered as a maladaptive strategy that leads to increased stress (Bettis et al., 2016; Ding et al., 2021). We thus hypothesized that: approach and acceptance coping styles would be associated with lower re-entry stress (Hypotheses 1a and 1b, respectively), and avoidance coping style would be related to higher re-entry stress (Hypothesis 1c).

### The Sociocultural Context of the Home Country

Importantly, the extent to which people experience re-entry stress is not only a function of individual differences in coping styles but is also influenced by contextual factors. It has been established that contextual or societal characteristics, such as cultural values, can have a considerable impact on cross-cultural adaptation (Ward & Geeraert, 2016). Geeraert and colleagues (2019), for example, found that students from “tighter” cultures, that is, cultures with stronger social norms, are better able to adapt to a new culture compared with individuals from loose cultures. Furthermore, findings by Güzel and Glazer (2019) indicated that students from cultures high in uncertainty avoidance (reluctance to tolerate unpredictability) and power distance (supportive of social hierarchies) experienced more difficulties with adaptation to a host culture. Researchers have thus clearly demonstrated that the values of the heritage culture are critical for understanding difficulties with cross-cultural transitions. However, to the best of our knowledge, no research to date has quantitatively explored the role of heritage cultural values in the re-entry process. We address this gap in the literature by exploring how country-level characteristics predict re-entry stress in high school student sojourners from 45 different countries.

In the present research, we use the most up-to-date theoretical framework and classification of cultural values of countries around the world based on data gathered in 2015–2016 (Minkov, 2018). The evidence suggests that cultural differences can be summarized by two main cultural dimensions, collectivism/individualism (hereon collectivism) and flexibility/monumentalism (hereon flexibility). Monumentalism describes societies that encourage an invariant, consistent self across different situations while in flexible societies, people tend to adjust themselves to specific situations. This cultural dimension reflects the East-West geographic axis in a sense that countries in the East generally score higher in flexibility (e.g., in East Asia) while countries in Africa and Latin America generally score higher in monumentalism (Minkov et al., 2018). Collectivism, however, encompasses differences in societal restrictiveness, support for social hierarchies, and adherence to traditional norms. It reflects cultural differences along the North-South geographic axis with Northwestern European countries generally scoring high on individualism and African countries scoring highest on collectivism (Minkov et al., 2017; Van de Vliert et al., 2024). It is worth noting that this conceptualization importantly differs from independent vs. interdependent aspect of self-construal (Krys et al., 2022; Markus & Kitayama, 1991; Vignoles et al., 2016). Terms such as individualism and collectivism are often used to describe differences in self-reliance and in interdependent nature of self and others. However, new evidence suggest that the self-construal aspect of culture may not be related to collectivism (Minkov et al., 2017, 2018).

We hypothesized that participants experience greater re-entry stress when they return to a home culture that is high in collectivism (Hypothesis 2a). We expect this relationship because such societies have stricter norms, stronger expectations to follow traditional values, and more pressure to act according to one’s role in the society (Gelfand et al., 2011; Minkov et al., 2017), which puts more pressure on an individual and can lead to increased stress. The relationship between flexibility and re-entry stress is less clear. Minkov and colleagues (2018) suggest that people from monumentalist countries have greater difficulty adapting their behavior compared with people from flexible countries, who are generally humbler and more adaptable. The adaptability theory suggests that individuals with lower capacity to regulate their psycho-behavioral functions when confronted with new or uncertain situations exhibit lower levels of well-being (Martin et al., 2012). Being less flexible in changing one’s own behavior may lead to greater re-entry difficulties upon return because one may need to adjust their behavior, cognition, values etc. to fit into their home society again. Alternatively, being less adaptive may lead to lower levels of cultural adaptation to the foreign culture in the first place. Lower levels of adaptation abroad were found to be related to lower levels of re-entry stress (Dykhouse & Bikos, 2019). This phenomenon may occur because fewer personal changes happened abroad, so less change is needed upon return to the home country. We thus posed an exploratory question about the relationship between flexibility and re-entry stress (Research Question 1).

Apart from home cultural values, we also explored two other characteristics of the home countries’ sociocultural environment. We examined how countries’ Human Development Index (United Nations Development Programme [UNDP], 2022) and cultural heterogeneity relate to re-entry stress. Homogeneity, as opposed to cultural diversity, refers to the extent to which individuals within a country share similar cultural backgrounds and values. Previous studies suggest that people who are less exposed to diversity tend to have a higher aversion to uncertainty and ambiguity (Kruglanski et al., 2002). In studies focusing on migrants returning to their homogeneous home country after living abroad (Ralph, 2012), returnees reported experiencing unwelcoming comments and being treated as “different” by local nationals. This reaction may be due to returnees standing out for their different beliefs or behaviors, combined with the local nationals’ discomfort with ambiguity. Such unwelcoming treatment can lead to greater stress upon returning to one’s home country. We thus hypothesized that greater homogeneity of the home country would be related to greater re-entry stress (Hypothesis 2b).

The Human Development Index is composed of three indicators: life expectancy, education, and GNI per capita. It is considered to reflect the country’s economic and social well-being. According to the social determinants of health framework (Braveman & Gottlieb, 2014; World Health Organization, 2023) as well as the stress process model proposed by Pearlin (1999), experiencing a lack of material resources, health facilities, education opportunities, and low income or unstable economic situation can contribute to greater stress and worse health outcomes. We therefore expected that lower HDI of the home country would be related to greater re-entry stress (Hypothesis 2c).

### The Interplay Between Coping Styles and Country-Level Characteristics

Complex processes such as reacculturation are not likely to be predicted and explained only by either individual differences or the cultural context, but, instead, these factors are likely to interact to predict outcomes (Geeraert et al., 2019). Some studies suggest that the tendency to use certain coping styles is culture-dependent (Szabo et al., 2017). In addition, cultural values may moderate the effectiveness of specific coping styles in response to stress (Kuo, 2011). Therefore, our study explored the moderating role of cultural context in the relationship between coping styles and re-entry stress. In essence, we investigated whether the association between coping styles and re-entry stress functioned differently in different cultures.

We expected individualism to amplify the impact of approach coping on reducing re-entry stress (Hypothesis 3a). We made this prediction based on previous claims that people from individualist cultures are prone to address the stressor directly rather than engage in passive coping (Shekriladze et al., 2021). Approach coping may thus be expected in an individualist cultural environment and may also serve as an adaptive response. We did not have specific predictions about the moderating role of flexibility, HDI, and heterogeneity on the relationship between approach coping and re-entry stress due to a lack of literature on this topic (Research Questions 2, 3, and 4, respectively).

Furthermore, we expected collectivism and flexibility to amplify the positive impact of acceptance coping on reducing re-entry stress (Hypotheses 3b and 3c, respectively). Several studies suggest that acceptance coping is especially prevalent and effective in collectivist countries (Morling et al., 2002), which is explained by collectivist persons’ tendency to find meaning in stressful experiences (Ji et al., 2022). Flexibility is characterized by long-term orientation values such as perseverance, humility, and focusing on the future (Minkov et al., 2018). Therefore, accepting a situation might be seen as the most effective strategy to deal with a stressor in the long-term in countries high in flexibility. We posed exploratory research questions about the moderating role of the HDI and heterogeneity with no specific predictions (Research Questions 5 and 6).

We expected individualism and flexibility to exacerbate the negative effect of avoidance coping on re-entry stress (Hypothesis 3d and 3e, respectively). This prediction was based on previous research showing that avoidance was related to greater stress for individuals scoring low on the allocentrism scale (i.e., person-level individualism), whereas avoidance was not related to greater stress for individuals scoring high on allocentrism (i.e., person-level collectivism; Shen, 2016). Moreover, avoidance coping has been considered an effective strategy in the short-term and with uncontrollable stressors (Holahan et al., 2017). It may be that this strategy is not seen as maladaptive in monumentalist countries where the focus is on the near future and immediate gratification. With regards to the moderating roles of HDI and heterogeneity we again made no specific predictions (Research Questions 7 and 8).

### The Aim of This Study

To summarize, the goals of the present paper were to answer the following questions: (1) What are the relationships between different types of coping styles and re-entry stress? (2) What are the relationships between country-level factors and re-entry stress? (3) Do country-level factors moderate the relationships between coping style and re-entry stress and how? The visualization of the planned analysis is presented in Figure 1 with Level 1 depicting self-reported time-varying variables and Level 2 depicting time-invariant country indices.

**Figure 1.**
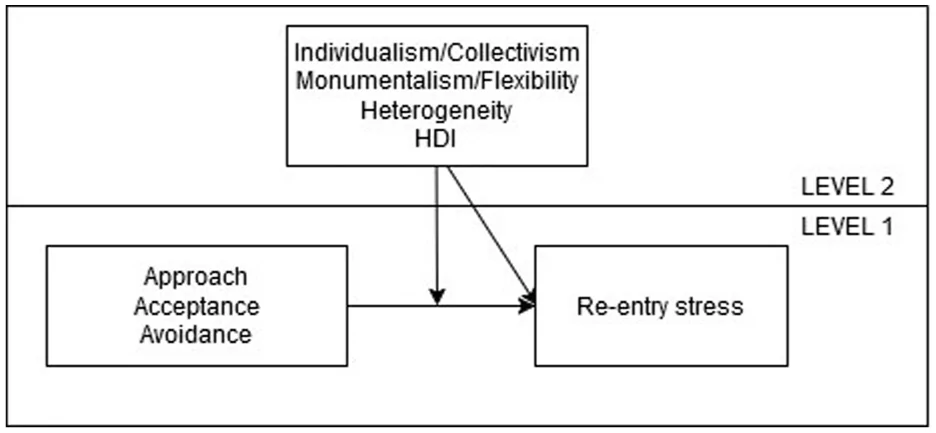
Visualization of the Planned Analysis *Note*. Level 1 = self-reported time-varying variables; Level 2 = time-invariant country indices.

Most research on re-entry stress relies on cross-sectional studies, which pose a significant limitation. Cross-sectional studies capture a single point in time, making it difficult to discern whether an individual’s high stress levels upon returning home are due to a genuine increase in stress or an inherently high sensitivity to stress. This distinction is essential since individuals differ in their baseline stress levels and stress sensitivity (Ebner & Singewald, 2017; Weyn et al., 2022). Only longitudinal data can thus accurately capture the *change* in stress levels and account for individual differences in stress sensitivity by tracking changes in stress levels over time. This study will add to the existing literature by analyzing longitudinal data and controlling for students’ baseline levels of stress before they went abroad and before they returned home.

## Method

### Research Design

In this study, secondary data from 2,480 study-abroad high school students were analyzed. Data were collected in 2012 as part of a research project named The Impact of Living Abroad (Geeraert, 2017). It was a longitudinal study with nine timewaves over a period of 18 months spanning from 2 months before going abroad to 6 months after the end of the exchange program. Two timewaves of measurement occurred before students went abroad (t1, t2), four timewaves while staying in a foreign country (t3, t4, t5, t6) and the last three timewaves occurred after the students returned to their home country (t7, t8, t9). We used re-entry data as the chief outcomes of this study. Re-entry data collection occurred approximately 2 weeks (t7), 2 months (t8), and 6 months (t9) after students returned home. The longitudinal nature of the data is especially valuable because we were able to control for baseline levels of stress and examine the revisualized effect of re-entry on stress levels.

### Sample Characteristics

A total of 2,480 sojourners participated in The Impact of Living Abroad study. Our analysis required that participants have at least one out of three re-entry stress scores to be included, resulting in a final sample of 1,485 young people. Participants were high school students (*M*_age_ = 17 years, *SD*_age_ = 0.97, 74% female) who participated in an international exchange program via a non-profit, volunteer-based organization called AFS Intercultural Programs. Students’ stay abroad lasted between 8 and 10 months. During this time, students enrolled at a local high school near their host family. Participants came from 45 different home countries and stayed abroad in one of the 51 host countries. A breakdown of sample size per country of origin alongside the countries’ flexibility, collectivism, HDI, and heterogeneity scores is shown in Table 1. Given the sample size of *N* = 1,485 and significance level α = .05, a post hoc power analysis using G*Power version 3.1.9.7 (Faul et al., 2007) suggested that obtained power was greater than 0.96 to detect small (*f²* = .02) effect sizes (J. Cohen, 2013).

**Table 1:** Sample Size Per Home Country with Country-Level Indices

Home Country	N	%	GCI	Flexibility	HDI 2012	Heterogeneity
Argentina	12	0.8	−0.37	−80	.843	.255
Australia	7	0.5	−1.17	41	.930	.093
Austria	39	2.6	−1.16	−2	.906	.107
Belgium	46	3.1	−1.16	63	.915	.555
Bolivia	1	0.1	0.52	−116	.674	.740
Brazil	60	4.0	−0.06	−83	.723	.541
Canada	9	0.6	−1.10	31	.921	.712
Chile	43	2.9	−0.14	−153	.824	.186
China	29	2.0	−1.15	134	.709	.154
Colombia	13	0.9	0.15	−182	.734	.601
Costa Rica	16	1.1	−0.25	−31*	.786	.237
Czech Republic	5	0.3	−1.12	17	.874	.322
Denmark	13	0.9	−1.37	51	.931	.082
Dominican Republic	5	0.3	−0.03	−154	.718	.429
Ecuador	9	0.6	0.23	−102	.751	.655
Finland	32	2.2	−1.30	71	.915	.132
France	57	3.8	−1.17	57	.882	.103
Germany	214	14.4	−1.35	46	.933	.168
Ghana	6	0.4	0.30	−205	.592	.673
Honduras	3	0.2	0.72	−74*	.599	.187
Hong Kong	18	1.2	−0.78	199	.918	.062
Hungary	11	0.7	−0.96	−9	.831	.152
Iceland	1	0.1	−0.88	45	.921	.080
India	11	0.7	0.25	17	.598	.418
Indonesia	26	1.8	0.67	−78	.678	.735
Italy	276	18.6	−0.73	−36	.883	.115
Japan	33	2.2	−1.18	234	.905	.012
Malaysia	6	0.4	0.01	−1	.780	.588
Mexico	21	1.4	0.09	−104	.760	.542
Netherlands	1	0.1	−1.29	87	.927	.105
New Zealand	39	2.6	−1.11	37	.924	.397
Norway	68	4.6	−1.33	36	.946	.059
Panama	3	0.2	0.05	−61*	.785	.553
Paraguay	6	0.4	0.74	−146*	.702	.169
Peru	1	0.1	0.29	−187	.742	.657
Philippines	3	0.2	0.67	−4	.685	.239
Portugal	2	0.1	−0.96	−24	.836	.047
Russia	3	0.2	−0.73	48	.811	.245
Spain	8	0.5	−0.90	2	.874	.417
Sweden	7	0.5	−1.43	21	.911	.060
Switzerland	42	2.8	−1.15	−21	.945	.531
Thailand	180	12.1	−0.14	87	.746	.634
Turkey	28	1.9	0.04	−52	.769	.320
US	66	4.4	−1.18	11	.916	.490
Venezuela	6	0.4	−0.06	−207	.767	.497
**Total**	**1,485**	**100.0**				

*Note. N* = sample size; GCI = Collectivism; * = imputed scores.

### Measures and Control Variables

Over 20 constructs were measured in the original study, but in this paper we focused on those relevant to our hypotheses and research questions. These measures include measures of stress, coping styles, and demographic information. Online surveys were conducted in 10 different languages. The measures were translated from English using the standard forward-back translation procedure.

#### Re-Entry Stress

Re-entry stress was assessed by a short version of the Perceived Stress Scale (Cohen et al., 1983) with 4 items rated on a 7-point Likert-type scale (1 = *never to* 7 = *always*). Higher scores indicated greater stress. Perceived stress was measured at all nine timewaves (t1-t9) over the period of 18 months by asking participants *“In the last 2 weeks how often have you felt. . .”* followed by items such as *“you were unable to control the important things in your life.*” Timewaves t7, t8, and t9 were operationalized as re-entry stress (outcome variable) because they occurred after the student returned home. The scale’s reliability at each timewave was acceptable (all αs > .70).

#### Baseline Levels of Stress

Perceived stress measured at timewaves t1-t6 was operationalized as baseline levels of stress (control variable). We examined the internal reliability in perceived stress between t1-t2 (before going abroad) and t3-t6 (while abroad) by computing Cronbach’s alpha. Stress scores were internally consistent across timewaves before going abroad and while staying abroad (both *α*s > .80), hence we computed one mean that represents perceived stress before going abroad (baseline stress 1) and one mean for perceived stress while staying abroad (baseline stress 2).

#### Coping Styles

Coping styles were measured by the Brief COPE scale (Carver, 1997) on a 7-point Likert-type scale (1 = *never* to 7 = *always*). This scale was administered at three timewaves in the study: 1 month before going abroad (t2), 2 months after arriving in the host country (t4) and 2 months after returning back to the home country (t8). We used the responses from t8 in our analyses, as they co-occurred with the assessment of re-entry stress. The Brief COPE assesses 16 different coping strategies: acceptance, active coping, disengagement, denial, distraction, humor, planning, reframing, religion, self-blame, substance abuse, emotional support (close and distant), instrumental support (close and distant), and venting. Participants were asked to “Think about any difficult times you have experienced since you arrived back in [name of the home country]. How often do you have the following reactions?.” In this study we focused on six strategies: active coping (e.g.; “I take action to try to make the situation better”), planning (e.g., “I think hard about what steps to take”), positive reframing (e.g., “I look for something good in what is happening”), acceptance (e.g., “I learn to live with it”), denial (e.g., “I refuse to believe that it has happened”), and behavioral disengagement (e.g., “I give up trying to deal with it”). Each strategy was measured by two items. Higher scores indicated more frequent use of a coping strategy.

#### Control Variables

Apart from baseline levels of stress, we controlled for gender, age, parents’ socioeconomic status (SES), prior sojourn experience, and third culture individuals. Parents’ SES was measured as an average of father’s and mother’s highest qualification where response 1 represented primary, 2 secondary and 3 higher qualification. Prior sojourn experience was measured by a single item “Have you ever spent longer than 1 month in another country?” with yes or no response. Third culture individuals were identified by a single item “Has the participant lived in their home country since birth?” with yes or no response.

### Country-Level Indices

Besides self-reported subject variables described above, four country-level scores/indices were obtained from published materials online.

#### Collectivism

The Global Collectivism Index (GCI) by Pelham and colleagues (2022) was used as a measure of collectivism. GCI was computed based on six indicators: fertility rate, living arrangement, marriage stability/divorce ratio, religiosity, collective transportation, and ingroup bias. Many different data sets were involved in their analysis, such as data from the World Values Survey (WVS), UNDP data, and Gallup WorldView data, gathered between 2002 and 2017. A lower score indicates lower collectivism (and greater individualist values) and a higher score indicates greater collectivism (and lower individualist values). GCI scores were available for all countries included in this data set.

#### Flexibility

Flexibility/Monumentalism scores were obtained from Minkov and colleagues (Minkov et al., 2018; Minkov & Kaasa, 2022). In their study, a large data set with a probabilistic sample gathered in 2015–2016 was used to compute country-level scores for 54 countries. Minkov and Kaasa (2022) later complemented the set of country scores by analyzing data from the WVS and provided additional scores, totaling 102 countries. A low score indicates lower flexibility (and higher monumentalism) and a high score indicates higher flexibility (and lower monumentalism) cultural values. Flexibility scores were available for 41 out of 45 countries included in this data set. For the remaining four countries, imputed scores were used (see Results).

#### Human Development Index

HDI represents a measure of a country’s well-being and standard of living. Higher HDI scores suggest greater country-level well-being. HDI scores for the year of 2012 were used in the analysis (UNDP, 2013) to match the time of survey data collection. HDI scores were available for all countries included in this data set.

#### Heterogeneity

We used the Fractionalization index by Alesina and colleagues (2002) as a measure of countries’ cultural heterogeneity. Alesina and colleagues presented three indices based on countries’ ethnic, language, and religious fractionalization using data from 1981 to 2001. In our study, we included the index of ethnic fractionalization. The larger the index, the greater the ethnic heterogeneity of the country. Heterogeneity scores were available for all countries included in this data set.

## Results

### Data Preparation and Missing Data

Out of the total 2,480 participants in this 18-month-long longitudinal study, 1,485 had at least one re-entry stress score and were suitable for the following analyses. About 1,140 participants completed the final assessment (t9) and 820 completed the survey at all nine timewaves, meaning some participants were missing certain data. Before imputing missing data, we examined missingness patterns. Little’s MCAR test showed data were not missing at random for individual-level variables as well as for country-level variables. We then explored the nature of the missing data by examining the association between attrition and demographic variables, perceived stress levels, and country indices.

Attrition was computed as a cumulative score of the number of completed timewaves for each participant. We assigned a value of +1 to participants who completed timewave 1, then adding +2 for completed t2, adding of +3 for completed t3 etc. Patterns of missing data due to attrition were then explored by correlating the attrition variable with demographic variables, perceived stress levels, and country indices. Results showed no significant correlations with demographic variables. There were several very small but statistically significant correlations at *p* < .001 level with HDI (*r* = .14) and collectivism (*r* = −.10), and at *p* < .05 level with stress (*r* = −.08) and third culture individuals (*r* = .06). All effect sizes were *d* < 0.02 which means that less than 2% of attrition could be explained by each of these variables. Overall, although several significant correlations were found, differential attrition occurred at a low level. Therefore, although we failed to establish that data were missing completely at random (MCAR), we concluded that attrition was missing at random (MAR; Johnson & Young, 2011).

Before data imputation, we also scanned for out-of-range values. All values were within expected ranges (e.g., 1–7 on a Likert-type scale). The exception was “Time of survey completion upon re-entry.” Time was recorded in days anchored on participants’ return travel date (i.e., how many days have passed since returning to the home country until completing the survey?). Upon visual inspection of the Time variable, we noticed four participants who had negative values, so we dropped them from further analysis. Several extreme values in the positive direction were noted, with a few participants completing the t9 survey considerably later (maximum = 531 days) than most participants (median = 199 days). To avoid the issue of outliers, we recoded the values over 365 days to exactly 365 days (corresponding to 1 year). According to previous findings, re-entry stress levels return to baseline stress levels within one year of re-entry (Dykhouse & Bikos, 2019). Therefore, it is reasonable to assume that survey responses at one year after re-entry or at 17 months after re-entry would not be considerably different.

Data were imputed in two groupings: for individual-level variables and country-level variables, using the Expectation-Maximization (EM) method. This method was found to be the most appropriate estimation method for several reasons. First, literature suggests EM imputation is suitable for data that are not MCAR (Sportisse et al., 2024). Second, compared with Multiple Imputation (MI), EM is robust for retaining the same variance-covariance structure despite non-normal multivariate distribution (Noghrehchi et al., 2021). And third, EM yields a new discrete data set, which can then be used for all further analysis (unlike, for example, FIML).

We were able to obtain HDI, GCI, and homogeneity scores for all countries included in this data set. Flexibility scores, however, were not available for four countries. In total, 1.8% of the participants were missing the flexibility score (because their country was not included in the rating exercise), which was imputed with EM. The imputed scores were then carefully examined in relation to relevant literature and similarity to other countries for which we had valid scores (Minkov et al., 2018; Minkov & Kaasa, 2022).

#### Exploratory Factor Analysis (EFA) on the Brief COPE Scale

EFA was conducted to examine the factorial structure of the scale and to reduce the number of variables into fewer predictors. We aimed to determine whether the items loaded coherently onto factors consistent with theoretical expectations described in the Literature review and previous analysis on a different subset of the same data set (Demes & Geeraert, 2015). We expected active coping and planning items to load on a single factor, and similarly for disengagement with denial, and acceptance with positive reframing items. We did not have predictions for the emotional support, instrumental support, substance abuse, and self-blame strategies.

We first evaluated internal reliabilities and external validities of the 16 strategies by assessing the Spearman-Brown (SB) coefficient and bivariate correlations between coping strategies and re-entry stress. Eisinga and colleagues (2013) demonstrated that the SB coefficient is a good alternative to Cronbach’s alpha for assessing internal reliabilities of very short scales, particularly those with only two items. Results revealed that internal consistency was low for venting (*SB* = .35) and distraction (*SB* = .48). Furthermore, correlations with re-entry stress were particularly low for religion (*r* = .01), humor (*r* = −.05), distant emotional support (*r* = .04), and distant instrumental support (*r* = .06). We decided to remove these six coping strategies, and continued with the analysis on the 10 remaining strategies with acceptable reliability (*SB*s between .57 and .90) and external validity (*r*s between ±.12 and .45).

Bartlett’s test (χ^2^ = 13279.84, *df* = 190, *p* < .001) and the Kaiser–Meyer–Olkin (KMO) measure of sampling adequacy (KMO = 0.82) indicated it was appropriate to conduct EFA on these data. The scree plot and eigenvalues suggested that the 20 items were best summarized by six components. EFA using the varimax rotation revealed that the higher-order factor of the approach coping style included active coping and planning items (*SB* = .74), avoidance coping style included denial and behavioral disengagement items (*SB* = .77), acceptance coping style included positive reframing and acceptance items (*SB* = .68), and social support included close emotional and instrumental support items (*SB* = .93). Self-blame (*r* = .54) and substance abuse (*r* = .82) were maintained as separate strategies.

The EFA resulted in six higher-order factors that provided a more simplified and efficient conceptualization of coping styles than the original 16 factors. Nevertheless, the goals of the study concerned just three of these six coping styles, namely the approach, acceptance, and avoidance coping styles which were utilized in subsequent analyses, so these three were brought forward for further analysis.

### Statistical Model

The data were analyzed through a series of multilevel models using R Statistical software, version 4.4.1 (2024-06-14 ucrt; R Core Team, 2024). Models were built in a systematic and sequential manner as proposed by Brauer and Curtin (2018), where random effects of the model are included first, and fixed effects are added subsequently. Before any analyses were conducted, predictor variables were standardized for easier comparison and interpretation of beta coefficients.

In the first step, we were interested in whether there was a need for a three-level hierarchical data structure to be modeled, where time was nested within participants and participants were nested within countries. The intraclass correlation (ICC) showed that 37% of the variance in the outcome was at the time-varying level (within-person variance), 62% at the person level (between-person variance), and 4% of the variance was at the country-level (between-countries variance). These results show that there was a need for a hierarchical data structure where time was nested within participants (two levels). In contrast, the need for a three-level hierarchical data structure was not supported because only a very small proportion of the total variance in the outcome was attributable to differences between countries (Koo & Li, 2016). Re-entry stress was therefore analyzed in a series of two-level models with time as the primary unit of analysis (Level 1, *n* = 4,455) nested within persons (Level 2, *n* = 1,481).

The second step involved adding control variables to the model; time, sex, age, parent’s socioeconomic status, previous sojourner experience, third culture individuals, and baseline levels of stress. Time was a significant predictor of re-entry stress (*p* = .023), although time alone accounted for a very small portion (0.05%) of within-subject variation. Nevertheless, the variable of time was retained as a control variable in all subsequent models. Furthermore, female students reported greater re-entry stress than male students (*p* = .004), but no other demographic control variable reached significance (all other *p*s > .37). Gender was retained as a control variable in subsequent models. Baseline stress 1 (before going abroad) and baseline stress 2 (while staying abroad) significantly predicted re-entry stress (*p*s < .001). Students with higher baseline levels of stress before going abroad and while staying abroad reported greater re-entry stress. To distinguish between *change*s in stress levels due to cultural transition (as opposed to individuals generally having high stress levels), we kept baseline stress 1 and 2 as covariates in subsequent models, which resulted in the revisualization of the outcome variable. The inclusion of control variables in the model (Model 1, see Table 2) significantly improved the null model, χ^2^(4) = 914.7, *p* < .001, as demonstrated by a significantly decreased deviance score. Time, gender, baseline stress 1, and baseline stress 2 accounted for 34.82% of variance together. Building on these first two steps, we then continued with two separate models: the coping model and the country-level model.

**Table 2.** Results of the Multilevel Analysis Models of Coping Styles on Re-Entry Stress.

	Model 1			Model 2		
Predictor	β	*SE*	*p*	β	*SE*	*p*
Intercept	2.83	0.03	<.001	2.84	0.03	<.001
Time (linear)	0.02	0.01	.023	0.02	0.01	.023
Baseline stress 1	0.23	0.02	<.001	0.19	0.02	<.001
Baseline stress 2	0.42	0.02	<.001	0.33	0.02	<.001
Gender	0.11	0.04	.004	0.09	0.04	.005
Approach				−0.01	0.02	.598
Acceptance				−0.18	0.02	<.001
Avoidance				0.24	0.02	<.001
Model statistics	deviance = 10,051 χ^2^(4) = 914.7, *p* <.001			deviance = 9,775 χ^2^(3) = 275.7, *p* <.001		
ICC within	55.40%			61.98%		
ICC between	44.59%			38.02%		
Pseudo *R*^2^ for fixed effects	34.82%			41.76%		

*Note*. ICC = intraclass correlation.

#### Coping Model

To analyze the association between coping styles and re-entry stress, we added the three coping styles as fixed effects to the model. Contrary to our expectations, approach coping style did not yield a significant main effect on re-entry stress after controlling for other variables in the model (Hypothesis 1a; β = −.01, 95% CI= [−0.05, 0.03], *p* = .598). Acceptance coping style was negatively related to re-entry stress, indicating that the use of acceptance coping predicted lower re-entry stress (β = −.18, 95% CI = [−0.23, −0.14], *p* < .001). This result is consistent with Hypothesis 1b. Finally, avoidance coping style exerted a positive effect on re-entry stress, demonstrating that avoidance was predictive of greater re-entry stress (β = .24, 95% CI = [0.19, 0.27], *p* < .001). This finding was consistent with Hypothesis 1c. By comparing pseudo *R*^2^ of Model 1 and Model 2, which show how much variance is explained by the fixed effects in the models, we see that the three coping styles explained about 7% of unique variance over and above covariates (Model 2, see Table 2). Among all coping strategies, avoidance manifested the largest effect size. This model had a significantly better fit compared with the Model 1, χ^2^(3) = 275.7, *p* < .001, because it incorporated important covariates and three coping styles, which better explain the levels of re-entry stress.

#### Country-Level Model

To analyze the associations between characteristics of the home country and re-entry stress, we added collectivism, flexibility, HDI, and homogeneity as fixed effects to the model. The Variance Inflation Factor (VIF) revealed multicollinearity between collectivism and HDI (*r* = −.88, *p* < .01), so HDI was omitted from all subsequent models. High correlations between collectivism and countries’ social and economic indices are well established (Gelfand et al., 2011; Minkov & Kaasa, 2022; Pelham et al., 2022; Uz, 2015), so high VIF is not entirely surprising. The results of the analysis revealed that collectivism had a positive main effect on re-entry stress, in support of Hypothesis 2a (β = .05, 95% CI= [0.00, 0.09], *p* = .047). This result suggests that students who returned to countries higher in collectivism experienced more stress. Flexibility did not yield a significant main effect on re-entry stress after controlling for other variables in the model (β = −.02, 95% CI = [−0.05, 0.02], *p* = .352), answering Research Question 1. Furthermore, heterogeneity yielded a negative main effect on re-entry stress (β = −.07, 95% CI = [−0.11, −0.03], *p* = .002), showing that students returning to more homogeneous countries experienced greater re-entry stress. Thus, Hypothesis 2b was supported. The three country characteristics explained about 0.36% of unique variance over and above covariates (see Model 3, Table 3). Overall, heterogeneity had the largest effect size among all country-level variables. This model had a significantly better fit compared with Model 1 due to the inclusion of important covariates and country characteristics, which explained the levels of re-entry stress well, χ^2^(3) = 13.9, *p* = .003.

**Table 3.** Results of the Multilevel Analysis Models of Country Characteristics on Re-entry Stress.

	Model 1			Model 3		
Predictor	β	*SE*	*p*	β	*SE*	*p*
Intercept	2.83	0.03	<.001	2.82	0.03	<.001
Time (linear)	0.02	0.01	.023	0.02	0.01	.025
Baseline stress 1	0.23	0.02	<.001	0.24	0.02	<.001
Baseline stress 2	0.42	0.02	<.001	0.42	0.02	<.001
Gender	0.11	0.04	.004	0.12	0.04	.002
GCI				0.05	0.02	.047
Flexibility				−0.02	0.02	.352
Heterogeneity				−0.07	0.02	.002
Model statistics	deviance = 10051, χ^2^(4) = 914.7, *p* <.001			deviance = 10037 χ^2^(3) = 13.9, *p* =.003		
ICC within	55.40%			55.65%		
ICC between	44.59%			44.34%		
Pseudo *R*^2^ for fixed effects	34.82%			35.18%		

*Note*. GCI = Collectivism; ICC = intraclass correlation.

#### Interactions Between Coping Strategies and Country-Level Indices

In the last step, we investigated interactions between each of the three coping styles and four country-level variables: flexibility, collectivism, HDI and heterogeneity. We conducted a series of 12 multilevel models with time as a control variable. At the level of individual predictors, 11 out of 12 interaction effects did not reach statistical significance (see Tables 6, 7, and 8 for the summary statistics in the supplementary material). No interaction effects between acceptance coping and country-level variables were significant (flexibility, *p* = .625; GCI, *p* = .687; HDI *p* = .699; and heterogeneity, *p* = .738), suggesting that none of the country variables moderated the relationship between acceptance coping style and re-entry stress. Similarly, no interaction effects between avoidance coping and country-level variables were significant (flexibility, *p* = .205; GCI, *p* = .609; HDI, *p* = .755; and heterogeneity, *p* = .636) showing that none of the country variables moderated the relationship between avoidance coping style and re-entry stress. Interactions between approach coping and flexibility (*p* = .148), HDI (*p* = .108), and heterogeneity (*p* = .108), did not yield statistically significant findings, while the hypothesized interaction of approach and GCI did achieve statistical significance (β = −.05, 95% CI = [−0.10, −0.00], *p* = .037, see Table 4).

**Table 4.** Multilevel Analysis Model of the Interaction Effect Between Approach Coping and GCI.

Predictor	β	*SE*	*p*
Intercept	2.92	0.02	<.001
Time (linear)	0.02	0.01	.019
Approach	−0.31	0.03	<.001
GCI	0.09	0.02	<.001
Approach x GCI	−0.05	0.03	.037

*Note.* GCI = Collectivism.

However, the graphed result did not confirm our predictions. We expected individualism (low GCI) to amplify the impact of approach coping on reducing re-entry stress but the simple slope analysis revealed that collectivism amplified the negative slope of approach coping on re-entry stress. As shown in Figure 2, for participants who very frequently used an approach coping style, there were no differences in re-entry stress levels based on their home countries’ collectivism scores. However, among individuals not using approach coping, re-entry stress was highest for those from collectivist countries. To summarize, among the country-level variables, only GCI moderated the relationship between approach coping style and re-entry stress while flexibility, HDI, and heterogeneity did not.

**Figure 2.**
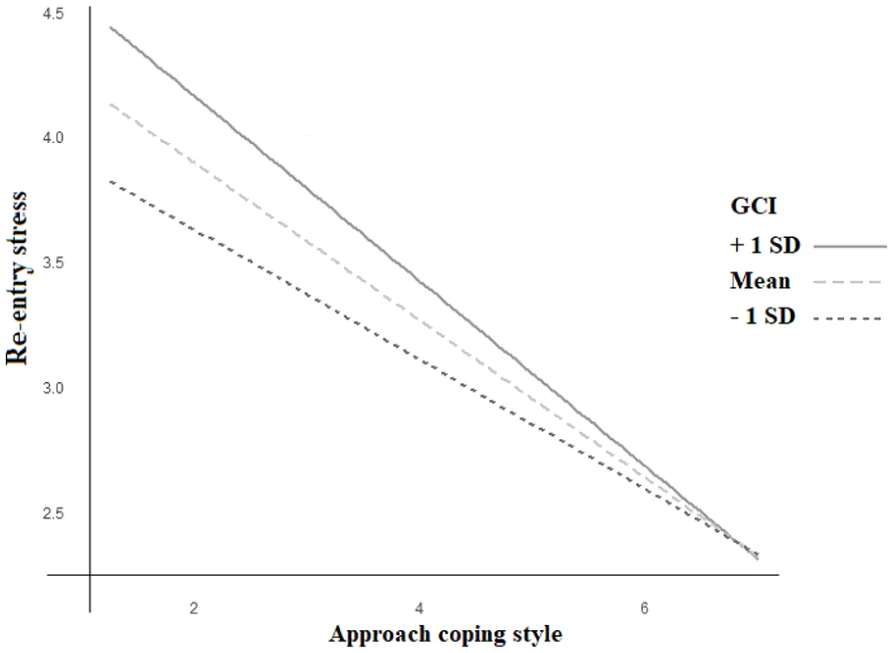
Collectivism Moderating the Effect of Approach Coping Style on Re-Entry Stress *Note. y*-axis = Re-entry stress on a 1–7 scale range; x-axis = Approach coping style on a 1–7 scale range; GCI = Collectivism.

### Exploratory Analyses: The Associations Among Coping Styles and Re-Entry Stress Over Time

To better understand temporal relationships among re-entry stress and coping styles, we examined how each of the three coping styles at t8 predicted change in stress from t8 to t9. The three separate models revealed that all three coping styles at t8 significantly predicted stress at t9 while controlling for stress at t8, but the results yielded small effect sizes. We found a negative relationship between stress and acceptance coping (β = −.09, 95% CI = [−0.11, −0.07], *p* < .001), a negative relationship with approach coping (β = −.09, 95% CI = [−0.11, −0.06], *p* < .001), and a positive relationship with avoidance coping style (β = .13, 95% CI = [0.10, 0.15], *p* < .001). These results suggest that greater use of acceptance and approach coping styles at 2 months after re-entry predicted lower levels of stress at 6 months after re-entry. Conversely, greater use of avoidance coping at 2 months predicted greater levels of stress at 6 months after re-entry.

Furthermore, we explored whether the change in coping styles over time predicted re-entry stress. We performed additional analysis, including previous waves of coping styles as control variables into the coping model. At the same time, controlling for previous waves of coping styles can illuminate whether the coping styles at t8 (which were the main predictors in the coping model) reflect coping efforts that are specific to the re-entry setting (i.e., re-entry coping styles), or participants’ general trait-like coping patterns that are grounded in personality (Guadalupe & DeShong, 2025). We thus added approach, acceptance, and avoidance coping styles assessed at t2 (before going abroad) and at t4 (while staying abroad) to the coping model (see Table 5).

**Table 5.** Results of the Multilevel Analysis Models of t2, t4, and t8 Coping Styles on Re-Entry Stress.

	Control Model			Coping Model		
Predictor	β	*SE*	*p*	β	*SE*	*p*
Intercept	2.83	0.03	<.001	2.85	0.03	<.001
Time (linear)	0.02	0.01	.023	0.02	0.01	.041
Baseline stress 1	0.22	0.02	<.001	0.20	0.02	<.001
Baseline stress 2	0.42	0.02	<.001	0.36	0.02	<.001
Gender	0.12	0.04	.004	0.09	0.04	.013
T2 Approach	0.03	0.02	.126	0.06	0.02	.006
T2 Acceptance	−0.06	0.02	.004	−0.02	0.02	.339
T2 Avoidance	0.07	0.02	.001	0.02	0.02	.432
T4 Approach	0.02	0.02	.405	0.02	0.02	.284
T4 Acceptance	0.01	0.02	.683	0.05	0.02	.008
T4 Avoidance	−0.02	0.02	.429	−0.06	0.02	.003
T8 Approach				−0.04	0.02	.029
T8 Acceptance				−0.17	0.02	<.001
T8 Avoidance				0.22	0.02	<.001
Model statistics	deviance = 10,071			deviance = 9,777 χ^2^(3) = 294, *p* <.001		
ICC within	55.93%			62.96%		
ICC between	44.07%			37.04%		
Pseudo *R*^2^ for fixed effects	35.21%			42.46%		

*Note*. ICC = intraclass correlation.

After controlling for prior waves of coping styles, approach (β = −.04, 95% CI = [−0.02, 0.06], *p* = .029), acceptance (β = −.17, 95% CI = [−0.20, −0.13], *p* < .001), and avoidance (β = .22, 95% CI = [−0.08, −0.00], *p* < .001) at t8 were significantly associated with re-entry stress, suggesting that increased use of acceptance and approach coping styles from earlier timewaves to re-entry period was related to lower re-entry stress, whereas increased use of avoidance coping was related to greater stress. Compared with the control model (containing only control variables), this model explained an additional 7% of variance (see Table 5). These results suggest that coping styles used specifically after re-entry (at t8) have a unique association with stress outcome, above and beyond individuals’ prior coping tendencies.

## General Discussion

This study aimed to address three critical gaps in the reacculturation literature. First, research to date has largely neglected the role of coping in reacculturation. To illustrate the importance of utilizing adaptive coping styles upon re-entry to the home culture, we examined the associations between approach, acceptance, and avoidance coping styles and re-entry stress. Second, whereas existing literature describes various intrapersonal and interpersonal factors predicting re-entry stress (Černigoj et al., 2024), no study to date has quantitatively explored the role of sociocultural context in the re-entry process. We thus examined how collectivism, flexibility, and cultural heterogeneity relate to re-entry stress. Third, we further broadened the scope of the reacculturation literature by introducing the interaction effects of intrapersonal factors (coping styles) and sociocultural factors (country-level characteristics) on re-entry stress.

Regarding the first research question, as expected, we found that the use of acceptance coping style predicted lower re-entry stress while avoidance coping style predicted greater re-entry stress. The use of an approach coping style, however, did not predict re-entry stress. Re-entry into the home culture can be perceived as a stressor that is difficult to control. Previous literature has found that emotion-focused coping, such as acceptance, is particularly effective for reducing stress when dealing with uncontrollable stressors, as it helps individuals adapt to the situation rather than attempt to change it (Göral et al., 2006; Szabo et al., 2016). In contrast, problem-focused coping styles like approach coping, may not be a particularly effective response in uncontrollable stressful circumstances (Main et al., 2011; Szabo et al., 2016). Avoidance coping has typically been considered as a maladaptive emotion-focused style that is associated with increased stress and reduced well-being in general (Ding et al., 2021; Lynch et al., 2001), and specifically among adolescents (Cicognani, 2011). Our findings showed that acceptance coping predicted reduced stress and avoidance predicted increased stress, in line with what has been found in the broader literature on coping with stress. In addition, the exploratory analysis showed that coping at the time of re-entry is uniquely important for stress outcomes, above and beyond earlier coping styles. This means that regardless of sojourners’ previous tendencies or general coping patterns, the coping styles they use during the re-entry period can influence their stress levels.

Regarding the second research question, we found that higher country-level collectivism and greater cultural homogeneity predicted greater re-entry stress. In collectivist societies, students may experience greater pressure to conform to traditional norms and social roles upon return and this can lead to greater re-entry stress (Minkov et al., 2017). Furthermore, returning to a culturally homogeneous environment may be challenging because such a society can be less accepting of differences and people standing out (Kruglanski et al., 2002; Ralph, 2012). Returnees, who often exhibit behaviors and beliefs shaped by more diverse contexts may therefore be perceived and treated as outsiders, leading to greater re-entry stress. Our findings also showed that when controlling for baseline levels of stress, flexibility did not predict re-entry stress. One explanation might be that the effect of flexibility on stress is mediated by a third individual difference variable, like sociocultural adaptation to the foreign culture. Individuals from monumentalist countries generally tend to have an invariant self and are less adaptable (Minkov et al., 2018). Being less adaptive may lead to lower levels of sociocultural adaptation while staying abroad, which is, in turn, related to lower re-entry stress upon return (Dykhouse & Bikos, 2019). Hence, it is possible that flexibility has indirect effects on re-entry stress through sociocultural adaptation to the foreign culture. Another possible explanation lies in the specific characteristics of our participants, such as their age. Previous research suggests that younger age is related to greater adaptive capacity to manage life challenges (Martin et al., 2012), therefore the results might have been different among older populations. Moreover, perhaps the influence of the home country’s characteristics, such as cultural values and socioeconomic well-being, are more distinct if a person resided in a host country with very different characteristics (e.g., returning home from a very monumentalist country to a flexible country or from an affluent country to a low-income country; Ward & Geeraert, 2016). Future research should thus include host country indices and explore the role of both cultural and economic differences between the host and home countries.

Answering the third research question about the joint effects of coping styles and country-level characteristics, country-level variables did not moderate the relationship between acceptance and re-entry stress and between avoidance and re-entry stress. However, we did find an interaction between approach coping and collectivism. Contrary to our predictions, our findings showed that not using approach coping was related to greater stress among students from collectivist countries compared with individualist countries in the re-entry setting. Previous literature suggests that individuals from individualist cultures are more likely to address the stressor directly than those from collectivist cultures (Persike & Seiffge-Krenke, 2016; Salash, 2013; Shekriladze et al., 2021). Based on this, we predicted that approach coping would be an expected, normative way of dealing with a stressor in an individualistic environment, serving as an adaptive response to the demands of the reacculturation process. This prediction rested on the assumption that the frequency of utilizing a coping style is related to its effectiveness. What we found, however, was that in collectivist countries, not using an approach coping style predicted greater re-entry stress. While this finding aligns with claims that collectivist cultures do not tend to address the stressor directly, such tendency may not be an adaptive response in the context of re-entry. A potential explanation lies in the restrictive nature of behavioral norms in collectivist societies. Upon returning, individuals may need to navigate these norms by engaging in more deliberate planning and actively managing the situation, which may explain why the lack of approach coping was related to greater re-entry stress in collectivist environments.

An additional finding emerged in this study that is worth nothing. When testing the hierarchical data structure for our multilevel model analysis, we found very little between-country variation in re-entry stress. This result implies that overall, there were minimal cross-national differences in stress perception upon re-entry, which speaks to the potential universality of the cultural transition experience. Our study thus illustrates greater cross-national similarities, rather than differences, in perceived stress due to cultural transition.

### Strengths and Limitations of This Study

Apart from addressing many pertinent gaps in the reacculturation literature, a notable strength of this study was its longitudinal design. The longitudinal design allowed us to control for baseline levels of stress, assess actual change in stress levels, and account for individual differences in stress sensitivity (Ebner & Singewald, 2017). Another notable advantage was including participants from 45 different home countries worldwide which made it possible to study country-level characteristics and their effects on re-entry stress in a diverse set of countries. However, the study included a weaker representation of low-income countries (our sample’s average HDI [0.81] was above the global HDI average [0.69]; UNDP, 2013), countries with high collectivism scores (our sample’s average GCI [−0.5] was below the global GCI average [0]; Pelham et al., 2022), and African countries. Another limitation is the issue of discrepancies in time of students’ self-report data collection and of country-level data collection. For example, we used the Fractionalization index (Alesina et al., 2002) as a measure of cultural heterogeneity which is based on data from 1981 to 2001. Thus, this index may or may not be an accurate representation of the cultural structure of countries in 2012, which is when the students’ data collection occurred. Our study was restricted to a very specific group of returnees of young age, who were supported by an organization, so their experiences might not be representative of other returnee groups. Future research should be extended to different returnee groups apart from student sojourners, like work-related expatriates from underrepresented world regions.

### Conclusion and Practical Implications

This study demonstrated the importance of the development of adaptive coping styles upon re-entry to the home culture and the importance of studying the reacculturation process within the sociocultural context in which it occurs. With the increase of international migration and temporary stays abroad, this research has important practical implications. Experiencing re-entry stress can be challenging and costly for a returnee, their social circle, their organization, and society. Re-entry training programs could support and encourage coping strategies that are effective in the re-entry context, such as acceptance coping. It could also address the issues related to strict social norms and homogeneous cultural environments that may aggravate re-entry stress.

## Supplemental Material

sj-docx-1-psp-10.1177_01461672251391059 – Supplemental material for The Longitudinal Examination of Associations Among Coping Styles, Sociocultural Context, and Re-Entry Stress After Study Abroad Supplemental material, sj-docx-1-psp-10.1177_01461672251391059 for The Longitudinal Examination of Associations Among Coping Styles, Sociocultural Context, and Re-Entry Stress After Study Abroad by Adela Černigoj, Paul E. Jose, Ágnes Szabó and Nicolas Geeraert in Personality and Social Psychology Bulletin

## Data Availability

The data are not publicly available. Requests should be directed at geeraert@essex.ac.uk.

## References

[bibr1-01461672251391059] Alesina A. F. Devleeschauwer A. Easterly W. Kurlat S. Wacziarg R. T. (2002). Fractionalization. SSRN Electronic Journal. 10.2139/ssrn.319762

[bibr2-01461672251391059] Alkhalaf K. (2019). Reverse acculturative stress among Saudi students returning from the United States to Saudi Arabia [Doctoral thesis, Howard University]. ProQuest Dissertations and Theses Global.

[bibr3-01461672251391059] Bettis A. H. Forehand R. McKee L. Dunbar J. P. Watson K. H. Compas B. E. (2016). Testing specificity: Associations of stress and coping with symptoms of anxiety and depression in youth. Journal of Child and Family Studies, 25(3), 949–958. 10.1007/s10826-015-0270-z PMC5546749 28794609

[bibr4-01461672251391059] Brauer M. Curtin J. J. (2018). Linear mixed-effects models and the analysis of nonindependent data: A unified framework to analyze categorical and continuous independent variables that vary within-subjects and/or within-items. Psychological Methods, 23(3), 389–411. 10.1037/met0000159 29172609

[bibr5-01461672251391059] Braveman P. Gottlieb L. (2014). The social determinants of health: It’s time to consider the causes of the causes. Public Health Reports, 129(1, Suppl. 2), 19–31. 10.1177/00333549141291S206 PMC3863696 24385661

[bibr6-01461672251391059] Carver C. S. (1997). You want to measure coping but your protocol’ too long: Consider the brief cope. International Journal of Behavioral Medicine, 4(1), 92–100. 10.1207/s15327558ijbm0401_6 16250744

[bibr7-01461672251391059] Černigoj A. Szabó Á. Jose P. (2024). Systematic literature review of factors influencing reacculturation after returning home from a stay abroad. International Journal of Intercultural Relations, 102, 102037. 10.1016/j.ijintrel.2024.102037

[bibr8-01461672251391059] Cicognani E. (2011). Coping strategies with minor stressors in adolescence: Relationships with social support, self-efficacy, and psychological well-being. Journal of Applied Social Psychology, 41(3), 559–578. 10.1111/j.1559-1816.2011.00726.x

[bibr9-01461672251391059] Cohen J. (2013). Statistical power analysis for the behavioral sciences. Routledge. 10.4324/9780203771587

[bibr10-01461672251391059] Cohen S. Kamarck T. Mermelstein R. (1983). A global measure of perceived stress. Journal of Health and Social Behavior, 24(4), 385. 10.2307/2136404 6668417

[bibr11-01461672251391059] Demes K. A. Geeraert N. (2015). The highs and lows of a cultural transition: A longitudinal analysis of sojourner stress and adaptation across 50 countries. Journal of Personality and Social Psychology, 109(2), 316–337. 10.1037/pspp0000046 PMC4507515 26191963

[bibr12-01461672251391059] Descartes R. (2017). Discourse on the method for conducting one’s reason well and for seeking the truth in the sciences. W. Ross MacDonald School Resource Services Library.

[bibr13-01461672251391059] Ding Y. Fu X. Liu R. Hwang J. Hong W. Wang J. (2021). The impact of different coping styles on psychological distress during the COVID-19: The mediating role of perceived stress. International Journal of Environmental Research and Public Health, 18(20), 10947. 10.3390/ijerph182010947 PMC8535725 34682693

[bibr14-01461672251391059] Dykhouse E. C. Bikos L. H. (2019). Re-entry friction: The curious effects of cultural dislocation on outcomes for global service learning returnees. International Journal of Intercultural Relations, 72, 96–108. https://doi.org/http://dx.doi.org/10.1016/j.ijintrel.2019.07.004

[bibr15-01461672251391059] Ebner K. Singewald N. (2017). Individual differences in stress susceptibility and stress inhibitory mechanisms. Current Opinion in Behavioral Sciences, 14, 54–64. 10.1016/j.cobeha.2016.11.016

[bibr16-01461672251391059] Eisinga R. Grotenhuis M. te. Pelzer B. (2013). The reliability of a two-item scale: Pearson, Cronbach, or Spearman-Brown? International Journal of Public Health, 58(4), 637–642. 10.1007/s00038-012-0416-3 23089674

[bibr17-01461672251391059] Faul F. Erdfelder E. Lang A.-G. Buchner A. (2007). G*Power 3: A flexible statistical power analysis program for the social, behavioral, and biomedical sciences. Behavior Research Methods, 39(2), 175–191. 10.3758/BF03193146 17695343

[bibr18-01461672251391059] Finnacord. (2018). Global expatriates: Size, segmentation and forecast for the worldwide market. https://www.finaccord.com/Home/About-Us/Press-Releases/Global-Expatriates-Size,-Segmentation-and-Forecas

[bibr19-01461672251391059] Gaw K. F. (2000). Reverse culture shock in students returning from overseas. International Journal of Intercultural Relations, 24, 83–104. 10.1016/S0147-1767(99)00024-3

[bibr20-01461672251391059] Geeraert N. (2017). The impact of living abroad: Stress, adaptation, and intergroup contact. 10.5255/UKDA-SN-850827

[bibr21-01461672251391059] Geeraert N. Li R. Ward C. Gelfand M. Demes K. A. (2019). A tight spot: How personality moderates the impact of social norms on sojourner adaptation. Psychological Science, 30(3), 333–342. 10.1177/0956797618815488 PMC6419235 30673368

[bibr22-01461672251391059] Geeraert N. Ward C. (2025). Sojourners returning home: Examining acculturative stress and well-being trajectories [Manuscript in preparation].

[bibr23-01461672251391059] Geeraert N. Ward C. Hanel P. H. P. (2022). Returning home: The role of expectations in re-entry adaptation. Applied Psychology: Health and Well-Being, 14(3), 949–966. 10.1111/aphw.12361 PMC9541004 35384307

[bibr24-01461672251391059] Gelfand M. J. Raver J. L. Nishii L. Leslie L. M. Lun J. Lim C. Duan L. Almaliach A. Ang S. Arnadottir J. Aycan Z. Boehnke K. Boski P. Cabecinhas R. Chan D. Chhokar J. Amato A. D. Ferrer M. Fischlmayr I. C. . . .Marquez P. (2011). Differences between tight and loose cultures: A 33-nation study. American Association for the Advancement of Science, 332(6033), 1100–1104. 10.1126/science.l203980 21617077

[bibr25-01461672251391059] Göral F. S. Kesimci A. Gençöz T. (2006). Roles of the controllability of the event and coping strategies on stress-related growth in a Turkish sample. Stress and Health, 22(5), 297–303. 10.1002/smi.1107

[bibr26-01461672251391059] Gray K. M. Savicki V. (2015). Study abroad reentry: Behavior, affect, and cultural distance. The Interdisciplinary Journal of Study Abroad, XXVI, 264–278. https://frontiersjournal.org/plugins/generic/pdfisViewer/pdf.js/we...al.org%

[bibr27-01461672251391059] Guadalupe C. DeShong H. L. (2025). Personality and coping: A systematic review of recent literature. Personality and Individual Differences, 239, 113119. 10.1016/J.PAID.2025.113119

[bibr28-01461672251391059] Gullahorn J. T. Gullahorn J. E. (1963). An extension of the U-curve hypothesis. Journal of Social Issues, 19(3), 33–47. 10.1111/j.1540-4560.1963.tb00447.x

[bibr29-01461672251391059] Güzel H. Glazer S. (2019). Demographic correlates of acculturation and sociocultural adaptation: Comparing international and domestic students. Journal of International Students, 9(4), 1074–1094. 10.32674/jis.v10i1.614

[bibr30-01461672251391059] Herman J. L. Tetrick L. E. (2009). Problem-focused versus emotion-focused coping strategies and repatriation adjustment. Human Resource Management, 48(1), 69–88. 10.1002/hrm.20267

[bibr31-01461672251391059] Holahan C. J. Ragan J. D. Moos R. H. (2017). Stress☆. In Reference module in neuroscience and biobehavioral psychology. Elsevier. 10.1016/B978-0-12-809324-5.05724-2

[bibr32-01461672251391059] International Organization for Migration. (2024). World migration report 2024. https://publications.iom.int/books/world-migration-report-2024

[bibr33-01461672251391059] Ji L.-J. Yap S. Khei Z. A. M. Wang X. Chang B. Shang S. X. Cai H. (2022). Meaning in stressful experiences and coping across cultures. Journal of Cross-Cultural Psychology, 53(9), 1015–1032. 10.1177/00220221221109552

[bibr34-01461672251391059] Johnson D. R. Young R. (2011). Toward best practices in analyzing datasets with missing data: Comparisons and recommendations. Journal of Marriage and Family, 73(5), 926–945. 10.1111/j.1741-3737.2011.00861.x

[bibr35-01461672251391059] Kartoshkina Y. (2015). Bitter-sweet reentry after studying abroad. International Journal of Intercultural Relations, 44, 35–45. 10.1016/j.ijintrel.2014.11.001

[bibr36-01461672251391059] Koo T. K. Li M. Y. (2016). A guideline of selecting and reporting intraclass correlation coefficients for reliability research. Journal of Chiropractic Medicine, 15(2), 155–163. 10.1016/j.jcm.2016.02.012 PMC4913118 27330520

[bibr37-01461672251391059] Kruglanski A. W. Shah J. Y. Pierro A. Mannetti L. (2002). When similarity breeds content: Need for closure and the allure of homogeneous and self-resembling groups. Journal of Personality and Social Psychology, 83(3), 648–662. 10.1037/0022-3514.83.3.648 12219860

[bibr38-01461672251391059] Krys K. Vignoles V. L. de Almeida I. Uchida Y. (2022). Outside the “cultural binary”: Understanding why Latin American collectivist societies foster independent selves. Perspectives on Psychological Science, 17(4), 1166–1187. 10.1177/17456916211029632 PMC9274794 35133909

[bibr39-01461672251391059] Kuo B. C. H. (2011). Culture’s consequences on coping. Journal of Cross-Cultural Psychology, 42(6), 1084–1100. 10.1177/0022022110381126

[bibr40-01461672251391059] Lazarus R. S. Folkman S. (1984). Stress, appraisal, and coping (Issue 14). Springer.

[bibr41-01461672251391059] Lynch T. R. Robins C. J. Morse J. Q. Krause E. D. (2001). A mediational model relating affect intensity, emotion inhibition, and psychological distress. Behavior Therapy, 32(3), 519–536. 10.1016/S0005-7894(01)80034-4

[bibr42-01461672251391059] Main A. Zhou Q. Ma Y. Luecken L. J. Liu X. (2011). Relations of SARS-related stressors and coping to Chinese college students’ psychological adjustment during the 2003 Beijing SARS epidemic. Journal of Counseling Psychology, 58(3), 410–423. 10.1037/a0023632 21574694

[bibr43-01461672251391059] Markus H. R. Kitayama S. (1991). Culture and the self: Implications for cognition, emotion, and motivation. Psychological Review, 98(2), 224–253. 10.1037/0033-295X.98.2.224

[bibr44-01461672251391059] Martin A. J. Nejad H. Colmar S. Liem G. A. D. (2012). Adaptability: Conceptual and empirical perspectives on responses to change, novelty and uncertainty. Australian Journal of Guidance and Counselling, 22(1), 58–81. 10.1017/jgc.2012.8

[bibr45-01461672251391059] Minkov M. (2018). A revision of Hofstede’s model of national culture: Old evidence and new data from 56 countries. Cross Cultural & Strategic Management, 25(2), 231–256. 10.1108/CCSM-03-2017-0033

[bibr46-01461672251391059] Minkov M. Bond M. H. Dutt P. Schachner M. Morales O. Sanchez C. Jandosova J. Khassenbekov Y. Mudd B. (2018). A reconsideration of Hofstede’s fifth dimension: New flexibility versus monumentalism data from 54 countries. Cross-Cultural Research, 52(3), 309–333. 10.1177/1069397117727488

[bibr47-01461672251391059] Minkov M. Dutt P. Schachner M. Morales O. Sanchez C. Jandosova J. Khassenbekov Y. Mudd B. (2017). A revision of Hofstede’s individualism-collectivism dimension: A new national index from a 56-country study. Cross cultural and strategic management, 24(3), 386–404. 10.1108/CCSM-11-2016-0197

[bibr48-01461672251391059] Minkov M. Kaasa A. (2022). Do dimensions of culture exist objectively? A validation of the revised Minkov-Hofstede model of culture with World Values Survey items and scores for 102 countries. Journal of International Management, 28(4), 100971. 10.1016/j.intman.2022.100971

[bibr49-01461672251391059] Morling B. Kitayama S. Miyamoto Y. (2002). Cultural practices emphasize influence in the United States and adjustment in Japan. Personality and Social Psychology Bulletin, 28(3), 311–323. 10.1177/0146167202286003

[bibr50-01461672251391059] Noghrehchi F. Stoklosa J. Penev S. Warton D. I. (2021). Selecting the model for multiple imputation of missing data: Just use an IC! Statistics in Medicine, 40(10), 2467–2497. 10.1002/sim.8915 PMC8248419 33629367

[bibr51-01461672251391059] Pearlin L. I. (1999). The stress process revisited (pp. 395–415). 10.1007/0-387-36223-1_19

[bibr52-01461672251391059] Pelham B. Hardin C. Murray D. Shimizu M. Vandello J. (2022). A truly global, non-WEIRD examination of collectivism: The Global Collectivism Index (GCI). Current Research in Ecological and Social Psychology, 3, 100030. 10.1016/j.cresp.2021.100030

[bibr53-01461672251391059] Persike M. Seiffge-Krenke I. (2016). Stress with parents and peers: How adolescents from 18 nations cope with relationship stress. Anxiety, Stress, & Coping, 29(1), 38–59. 10.1080/10615806.2015.1021249 25738813

[bibr54-01461672251391059] Presbitero A. (2016). Culture shock and reverse culture shock: The moderating role of cultural intelligence in international students’ adaptation. International Journal of Intercultural Relations, 53, 28–38. 10.1016/j.ijintrel.2016.05.004

[bibr55-01461672251391059] Ralph D. (2012). Managing sameness and difference: The politics of belonging among Irish-born return migrants from the United States. Social and Cultural Geography, 13(5), 445–460. 10.1080/14649365.2012.698747

[bibr56-01461672251391059] R Core Team. (2024). R: A language and environment for statistical computing. R Foundation for Statistical Computing.

[bibr57-01461672251391059] Salash M. (2013). Differences in perceived stress and coping strategies between Ukrainian and US college students [Doctoral dissertation, Brigham Young University]. ProQuest Dissertations and Theses Global.

[bibr58-01461672251391059] Shekriladze I. Javakhishvili N. Chkhaidze N. (2021). Culture related factors may shape coping during pandemics. Frontiers in Psychology, 12, Article 634078. 10.3389/fpsyg.2021.634078 PMC8170015 34093315

[bibr59-01461672251391059] Shen J. J. (2016). Collectivistic coping, allocentrism, and stress [Doctoral dissertation, California State University]. ProQuest Dissertations and Theses Global. https://www.proquest.com/openview/8a1518e5d9dde83c9f8286f48ee974a3/1?pq-origsite=gscholar&cbl=18750

[bibr60-01461672251391059] Sportisse A. Marbac M. Laporte F. Celeux G. Boyer C. Josse J. Biernacki C. (2024). Model-based clustering with missing not at random data. Statistics and Computing, 34(4), 135. 10.1007/s11222-024-10444-2

[bibr61-01461672251391059] Szabo A. English A. S. Zhijia Z. Jose P. Ward C. Jianhong M. (2017). Is the utility of secondary coping a function of ethnicity or the context of reception? A longitudinal study across Western and Eastern cultures. Journal of Cross-Cultural Psychology, 48(8), 1230–1246. 10.1177/0022022117719158

[bibr62-01461672251391059] Szabo A. Ward C. Jose P. E. (2016). Uprooting stress, coping, and anxiety: A longitudinal study of international students. International Journal of Stress Management, 23(2), 190–208. 10.1037/a0039771

[bibr63-01461672251391059] United Nations Development Programme. (2013). Human development report 2013: The rise of the South: Human progress in a diverse world. https://hdr.undp.org/content/human-development-report-2013

[bibr64-01461672251391059] United Nations Development Programme. (2022). Human development report 2021-22: Uncertain times, unsettled lives: Shaping our future in a transforming world. https://hdr.undp.org/content/human-development-report-2021-22

[bibr65-01461672251391059] Uz I. (2015). The index of cultural tightness and looseness among 68 countries. Journal of Cross-Cultural Psychology, 46(3), 319–335. 10.1177/0022022114563611

[bibr66-01461672251391059] Van de Vliert E. Pelham B. Vandello J. A. (2024). East-West beliefs challenged by North-South evidence about collectivism versus individualism. Journal of Environmental Psychology, 101, 102498. 10.1016/j.jenvp.2024.102498

[bibr67-01461672251391059] Vignoles V. L. Owe E. Becker M. Smith P. B. Easterbrook M. J. Brown R. González R. Didier N. Carrasco D. Cadena M. P. Lay S. Schwartz S. J. Des Rosiers S. E. Villamar J. A. Gavreliuc A. Zinkeng M. Kreuzbauer R. Baguma P. Martin M. . . .Bond M. H. (2016). Beyond the “East–West” dichotomy: Global variation in cultural models of selfhood. Journal of Experimental Psychology: General, 145(8), 966–1000. 10.1037/xge0000175 27359126

[bibr68-01461672251391059] Ward C. (2001). The ABCs of acculturation. In Matsumoto D. (Ed.), Handbook of culture and psychology (pp. 411–466). Oxford University Press.

[bibr69-01461672251391059] Ward C. Geeraert N. (2016). Advancing acculturation theory and research: The acculturation process in its ecological context. Current Opinion in Psychology, 8, 98–104. 10.1016/j.copsyc.2015.09.021 29506811

[bibr70-01461672251391059] Weyn S. Van Leeuwen K. Pluess M. Goossens L. Claes S. Bosmans G. Van Den Noortgate W. Lutin E. Bröhl A. S. Chubar V. Geukens F. Bijttebier P. (2022). Individual differences in environmental sensitivity at physiological and phenotypic level: Two sides of the same coin? International Journal of Psychophysiology, 176, 36–53. 10.1016/j.ijpsycho.2022.02.010 35301026

[bibr71-01461672251391059] World Health Organization. (2023). Social determinants of health. https://www.who.int/health-topics/social-determinants-of-health

