# Supplementary material for: The Longitudinal Examination of Associations Among Coping Styles, Sociocultural Context, and Re-Entry Stress After Study Abroad
Source: Pers Soc Psychol Bull. 2025 Dec 1;52(11):3937–52. doi: 10.1177/01461672251391059 (PMC13646404; doi:10.1177/01461672251391059)
Supplement: Supplementary file 1 [file sj-docx-1-psp-10.1177_01461672251391059.docx]

Supplementary Material

**Table 5.** *Results of the Multilevel Analysis Models of the Interaction Effects with Approach Coping Style*

|  | Model 4: Flexibility | | |  | Model 5: GCI | | |  | Model 6: HDI | | |  | Model 7: Heterogeneity | | |
| --- | --- | --- | --- | --- | --- | --- | --- | --- | --- | --- | --- | --- | --- | --- | --- |
| Predictor | *β* | *SE* | *p* |  | *β* | *SE* | *p* |  | *β* | *SE* | *p* |  | *β* | *SE* | *p* |
| Intercept | 2.91 | 0.02 | <.001 |  | 2.92 | 0.02 | <.001 |  | 2.92 | 0.02 | <.001 |  | 2.91 | 0.02 | <.001 |
| Time (linear) | 0.02 | 0.01 | .017 |  | 0.02 | 0.01 | .019 |  | 0.02 | 0.01 | .019 |  | 0.02 | 0.01 | .018 |
| Approach | -0.28 | 0.03 | <.001 |  | -0.31 | 0.03 | <.001 |  | -0.32 | 0.03 | <.001 |  | -0.29 | 0.03 | <.001 |
| Country variable | 0.09 | 0.02 | <.001 |  | 0.09 | 0.02 | <.001 |  | -0.12 | 0.02 | <.001 |  | 0.04 | 0.02 | .081 |
| Approach x Country variable | 0.03 | 0.02 | .148 |  | -0.05 | 0.03 | .037 |  | 0.04 | 0.03 | .109 |  | 0.04 | 0.03 | .109 |

Note. GCI = Collectivism; HDI = Human Development Index

**Table 6.** *Results of the Multilevel Analysis Models of the Interaction Effects with Acceptance Coping Style*

|  | Model 8: Flexibility | | |  | Model 9: GCI | | |  | Model 10: HDI | | |  | Model 11: Heterogeneity | | |
| --- | --- | --- | --- | --- | --- | --- | --- | --- | --- | --- | --- | --- | --- | --- | --- |
| Predictor | *β* | *SE* | *p* |  | *β* | *SE* | *p* |  | *β* | *SE* | *p* |  | *β* | *SE* | *p* |
| Intercept | 2.91 | 0.02 | <.001 |  | 2.92 | 0.02 | <.001 |  | 2.92 | 0.02 | <.001 |  | 2.91 | 0.02 | <.001 |
| Time (linear) | 0.02 | 0.01 | .017 |  | 0.02 | 0.01 | .019 |  | 0.02 | 0.01 | .020 |  | 0.02 | 0.01 | .018 |
| Acceptance | -0.36 | 0.03 | <.001 |  | -0.38 | 0.03 | <.001 |  | -0.38 | 0.03 | <.001 |  | -0.39 | 0.03 | <.001 |
| Country variable | 0.11 | 0.02 | <.001 |  | 0.08 | 0.02 | <.001 |  | -0.11 | 0.02 | <.001 |  | 0.08 | 0.02 | <.001 |
| Acceptance x Country variable | 0.01 | 0.02 | .625 |  | -0.01 | 0.03 | .687 |  | 0.01 | 0.03 | .699 |  | 0.01 | 0.03 | .738 |

Note. GCI = Collectivism; HDI = Human Development Index

**Table 7**. *Results of the Multilevel Analysis Models of the Interaction Effects with Avoidance Coping Style*

|  | Model 12: Flexibility | | |  | Model 13: GCI | | |  | Model 14: HDI | | |  | Model 15: Heterogeneity | | |
| --- | --- | --- | --- | --- | --- | --- | --- | --- | --- | --- | --- | --- | --- | --- | --- |
| Predictor | *β* | *SE* | *p* |  | *β* | *SE* | *p* |  | *β* | *SE* | *p* |  | *β* | *SE* | *p* |
| Intercept | 2.92 | 0.02 | <.001 |  | 2.91 | 0.02 | <.001 |  | 2.91 | 0.02 | <.001 |  | 2.92 | 0.02 | <.001 |
| Time (linear) | 0.02 | 0.01 | .021 |  | 0.02 | 0.01 | .024 |  | 0.02 | 0.01 | .024 |  | 0.02 | 0.01 | .022 |
| Avoidance | 0.49 | 0.02 | <.001 |  | 0.49 | 0.02 | <.001 |  | 0.49 | 0.02 | <.001 |  | 0.50 | 0.02 | <.001 |
| Country variable | 0.10 | 0.02 | <.001 |  | 0.06 | 0.02 | .003 |  | -0.08 | 0.02 | <.001 |  | 0.05 | 0.02 | .021 |
| Avoidance x Country variable | -0.03 | 0.02 | .205 |  | 0.01 | 0.02 | .609 |  | -0.01 | 0.02 | .755 |  | 0.01 | 0.02 | .636 |

Note. GCI = Collectivism; HDI = Human Development Index
